# Dairy Product Consumption and Bladder Cancer Risk in the Prostate, Lung, Colorectal, and Ovarian (PLCO) Cohort

**DOI:** 10.3389/fnut.2020.00097

**Published:** 2020-07-21

**Authors:** Xin Xu

**Affiliations:** Department of Urology, School of Medicine, The First Affiliated Hospital, Zhejiang University, Hangzhou, China

**Keywords:** dairy product, bladder cancer, PLCO, cohort, risk

## Abstract

Evidence has suggested that dairy product consumption lowers the risk of several cancers, but these benefits may not occur with bladder cancer. In a cohort of 101,721 subjects in the Prostate, Lung, Colorectal, and Ovarian (PLCO) Cancer Screening Trial, we analyzed the effects of dairy product intake on bladder cancer risk using Cox proportional hazards regression. After a median of 12.5 years of follow-up, 776 new cases of bladder cancer were identified. We found no statistically significant association between total milk intake and bladder cancer risk. The multivariate-adjusted hazard ratio (HR) of bladder cancer for participants in the highest category of total milk intake compared with those in the lowest category was 1.13 (95% CI: 0.92–1.40; *p* for trend = 0.436). Among individual dairy foods, no statistically significant association was observed for a broad range of dairy products, including whole milk, 2% milk, 1% milk, skim milk, yogurt, regular butter, low fat butter, regular cheese, low fat cheese, and no fat cheese. These associations were not modified by smoking status (*p* for interaction > 0.05). In conclusion, findings from this large prospective analysis do not support an inverse association between dairy product consumption and bladder cancer risk.

## Introduction

Bladder cancer is the 10th most common cancer worldwide, with an estimated 549,000 new cases and 200,000 deaths in 2018 (1, 2). The most well-established risk factors for bladder cancer are cigarette smoking and occupational exposure to aromatic amines and 4,4′-methylenebis (2-chloroaniline) (3). Less-established risk factors for bladder cancer include lack of physical activity (4), obesity (5), a history of urinary calculi (6), and chronic urinary tract infection (7).

Recently, several epidemiological studies have shown a potential inverse association between intake of dairy products and bladder cancer risk and a meta-analysis reported that yogurt consumption was significantly associated with a decreased risk of bladder cancer (8). However, the two prospective studies (9, 10) included in this meta-analysis have reported inconsistent results. Another meta-analysis by Bermejo et al. (11) also indicated a reduced risk of bladder cancer associated with medium consumption of total dairy products and with medium and high consumption of milk and fermented dairy products. However, a pooled analysis of 13 cohort studies by Acham et al. (12) failed to find a significant association between total or individual dairy products and bladder cancer risk. This study aims at contributing to this debate by considering the association between dairy product consumption and bladder cancer risk in the prostate, lung, colorectal, and ovarian (PLCO) cohort.

## Materials and Methods

### Data Sources

The PLCO study is a large population-based cancer screening trial designed to evaluate whether selected screening methods could reduce mortality from prostate, lung, colorectal, and ovarian cancer, as described previously (13). Briefly, 154,952 individuals aged 55–74 years were recruited via 10 centers in the United States between 1993 and 2001. Subjects were included in this study if they completed the baseline questionnaire and were cancer free before completion of the diet history questionnaire (DHQ). All participants provided written informed consent, and the study was approved by the Institutional Review Boards at the National Cancer Institute.

### Data Collection

The baseline questionnaire included self-reported information on demographics (e.g., age, sex, ethnicity, and education), smoking status, family history of cancer, and medical history. Dietary data were collected using the DHQ (14), which included the portion size and frequency of intake of 124 food items and supplement use during the past year. The amount of dairy product intake was calculated using the detailed analysis file output by DietCalc, which determines the gram amounts by sex and serving size using a nutrient database based on national dietary data (USDA's 1994–96 Continuing Survey of Food Intakes by Individuals, available from the USDA Food Surveys Research Group) (15).

### Ascertainment of Bladder Cancer

Study participants were mailed a questionnaire annually to screen cancer cases. All reports of bladder cancer were followed up and medical records were abstracted and reviewed for case ascertainment. In this analysis, bladder cancer case was defined according to International Classification of Diseases for Oncology Second Edition (ICD-O-2), codes C67.0-C67.9. Vital status was obtained by the administration of the Annual Study Update questionnaires, reports from relatives, friends, or physicians, and National Death Index searches.

### Statistical Analysis

Cox proportional hazards regression was used to estimate hazard ratios (HRs) and 95% confidence intervals (CIs). Models were adjusted for age (categorical), sex (male vs. female), race (White, Non-Hispanic vs. Other), body mass index at the time of enrollment (<25 vs. ≥25 kg/m^2^), education (≤ high school vs. ≥some college), smoking status (never vs. former ≤ 15 years since quit vs. former >15 years since quit vs. former year since quit unknown vs. current smoker ≤ 1 pack per day vs. current smoker >1 pack per day vs. current smoker intensity unknown), vegetable intake (continuous), fruit intake (continuous), tea intake (continuous), alcohol drinking status (never vs. former vs. current), total energy intake (continuous), randomization arm (intervention vs. control), family history of any cancer (yes vs. no), and marital status (married vs. not married). Missing values for covariates were treated as dummy variables in the models. *p* for trend was calculated using a continuous variable created from medians within quartiles of dietary products. Likelihood ratio tests were used to test violations of the Cox proportional hazards assumption and heterogeneity of associations. A restricted cubic spline model (16) with three knots (i.e., 10th, 50th, and 90th percentiles) was also used to examine the association between dietary total milk intake and bladder cancer risk. All statistical analyses were performed using the software STATA version 15 (Stata Corp, College Station, TX, USA). All tests were two-sided.

## Results

After a median of 12.5 years of follow-up, 776 new cases of bladder cancer were identified from the 101,721 individuals included in our study. Cases were older, were more often men, and were more likely to be current smokers or ex-smokers. Table 1 shows the main characteristics of included subjects with and without bladder cancer.

**Table 1:** Main characteristic of 101,721 subjects in the PLCO cancer screening trial.

**Variables**	**Non-cases (*n* = 100,945)**	**Cases (*n* = 776)**	***p*-value**
Age (years), mean ± SD	62.4 ± 5.3	64.1 ± 5.2	<0.001
Sex (*n*, %)			
Male	48,847 (48.4)	627 (80.8)	<0.001
Female	52,098 (51.6)	149 (19.2)	
Smoking status (*n*, %)			
Never	48,363 (47.9)	189 (24.4)	<0.001
Current	9264 (9.2)	131 (16.9)	
Former	43,305 (42.9)	456 (58.8)	
Missing	13 (0.0)	0 (0.0)	
Education (*n*, %)			
≤ High school	42,574 (42.2)	354 (45.6)	0.066
≥Some college	58,177 (57.6)	419 (54.0)	
Missing	194 (0.2)	3 (0.4)	
BMI (*n*, %)			
<25.0 kg/m^2^	33,522 (33.2)	217 (28.0)	0.008
≥25.0 kg/m^2^	66,099 (65.5)	549 (70.7)	
Missing	1324 (1.3)	10 (1.3)	
Race (*n*, %)			
White, Non-Hispanic	91,765 (90.9)	738 (95.1)	<0.001
Other	9143 (9.1)	38 (4.9)	
Missing	37 (0.0)	0 (0.0)	
Alcohol drinking status (*n*, %)			
Never	10,061 (10.0)	52 (6.7)	0.003
Former	14,650 (14.5)	105 (13.5)	
Current	73,368 (72.7)	604 (77.8)	
Missing	2866 (2.8)	15 (1.9)	
Total energy (kcal/day), median (IQR)	1608 (1222–2102)	1742 (1306–2255)	<0.001
Randomization arm (*n*, %)			
Intervention	51,440 (51.0)	364 (46.9)	0.025
Control	49,505 (49.0)	412 (53.1)	
Marital status (*n*, %)			
Married	78,972 (78.2)	639 (82.3)	0.006
Not married	21,790 (21.6)	134 (17.3)	
Missing	183 (0.2)	3 (0.4)	
Family history of any cancer (*n*, %)			
Yes	56,388 (56.0)	450 (58.1)	0.236
No	44,557 (44.0)	326 (41.9)	

*PLCO, prostate, lung, colorectal and ovarian; SD, standard deviation; BMI, body mass index; IQR, interquartile range*.

**Figure 1:**
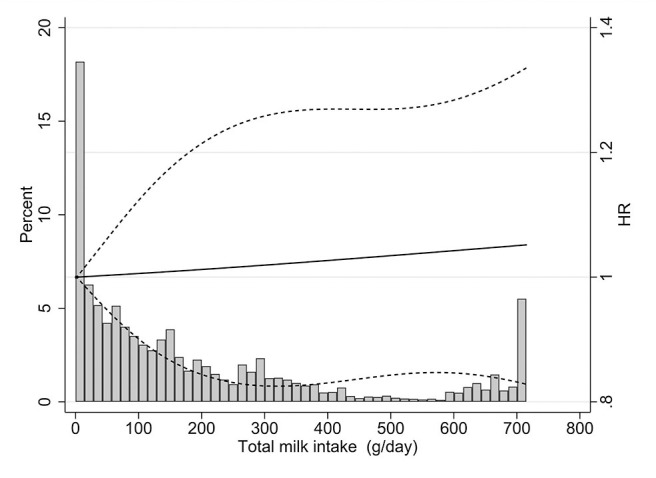
Dose-response analysis using restricted cubic spline model for the association between total milk intake and bladder cancer risk. Solid lines represent point estimates and dashed lines represent 95% confidence intervals. The histograms show the percentage of participants (left y axis) belonging to each level of total milk intake.

We found no statistically significant association between total milk intake and risk of bladder cancer (Table 2). The multivariate-adjusted HR of bladder cancer for participants in the highest category of total milk intake (525.87 g/day) compared with those in the lowest category (5.49 g/day) was 1.13 (95% CI: 0.92–1.40; *p* for trend = 0.243). A spline regression plot of bladder cancer risk in relation to total milk intake is shown in Figure 1. There was no statistical evidence for non-linearity (*p* for non-linearity >0.05). When stratifying by sex, the results were not substantially different. Total milk intake was not associated with bladder cancer risk either in male (HR_Q4vsQ1_ = 1.06, 95% CI = 0.84–1.34) or in female (HR_Q4vsQ1_ = 1.26, 95% CI = 0.80–1.98).

**Table 2:** Association between intake of dairy products and bladder cancer risk in the PLCO cancer screening trial.

**Variables (g/day)**	**Median (g/day)**	**Cohort (*n*)**	**Cases (*n*)**	**Age- and sex-adjusted HR (95% CI), *p*-value**	Multivariable adjusted HR (95% CI)^*^, *p*-value
Total milk					
Q1 (<30.02)	5.49	25,432	161	Reference group	Reference group
Q2 (≥30.02 to <115.88)	68.58	25,429	207	1.15 (0.93–1.41), *p* = 0.193	1.16 (0.94–1.42), *p* = 0.171
Q3 (≥115.88 to <285.49)	176.92	25,466	184	0.97 (0.78–1.19), *p* = 0.750	0.99 (0.80–1.22), *p* = 0.910
Q4 (≥285.49)	525.87	25,394	224	1.10 (0.90–1.35), *p* = 0.346	1.13 (0.92–1.40), *p* = 0.243
				*p* for trend = 0.590	*p* for trend = 0.436
Whole milk^#^					
Q1 (0)	0.00	90,451	688	Reference group	Reference group
Q2 (>0 to <21.47)	6.39	3854	16	0.59 (0.36–0.97), *p* = 0.036	0.58 (0.35–0.95), *p* = 0.031
Q3 (≥21.47 to <122.22)	58.78	3660	36	1.25 (0.89–1.75), *p* = 0.192	1.13 (0.81–1.60), *p* = 0.470
Q4 (≥122.22)	276.64	3756	36	1.09 (0.78–1.52), *p* = 0.627	0.97 (0.69–1.36), *p* = 0.860
				*p* for trend = 0.462	*p* for trend = 0.990
2% milk^#^					
Q1 (0)	0.00	70,212	510	Reference group	Reference group
Q2 (>0 to <40.39)	12.33	10,521	84	1.18 (0.93–1.48), *p* = 0.170	1.11 (0.88–1.40), *p* = 0.369
Q3 (≥40.39 to <161.04)	88.36	10,498	82	1.02 (0.81–1.29), *p* = 0.853	0.99 (0.78–1.25), *p* = 0.919
Q4 (≥161.04)	302.60	10,490	100	1.10 (0.89–1.36), *p* = 0.392	1.03 (0.83–1.29), *p* = 0.779
				*p* for trend = 0.484	*p* for trend = 0.880
1% milk^#^					
Q1 (0)	0.00	84,667	649	Reference group	Reference group
Q2 (>0 to <67.01)	22.33	5685	32	0.76 (0.53–1.09), *p* = 0.135	0.77 (0.54–1.10), *p* = 0.147
Q3 (≥67.01 to <223.74)	134.73	5725	41	0.84 (0.62–1.16), *p* = 0.292	0.87 (0.63–1.19), *p* = 0.390
Q4 (≥223.74)	395.22	5644	54	1.06 (0.80–1.40), *p* = 0.688	1.10 (0.83–1.46), *p* = 0.500
				*p* for trend = 0.873	*p* for trend = 0.641
Skim milk^#^					
Q1 (0)	0.00	59,591	478	Reference group	Reference group
Q2 (>0 to <77.63)	31.16	14,051	102	1.08 (0.87–1.34), *p* = 0.468	1.14 (0.92–1.42), *p* = 0.231
Q3 (≥77.63 to <261.65)	148.11	14,044	89	0.85 (0.68–1.07), *p* = 0.164	0.92 (0.73–1.15), *p* = 0.449
Q4 (≥261.65)	500.99	14,035	107	0.96 (0.78–1.19), *p* = 0.713	1.05 (0.85–1.30), *p* = 0.664
				*p* for trend = 0.522	*p* for trend = 0.866
Yogurt^#^					
Q1 (0)	0.00	41,006	420	Reference group	Reference group
Q2 (>0 to <5.42)	1.90	23,586	140	0.83 (0.68–1.00), *p* = 0.053	0.94 (0.77–1.14), *p* = 0.515
Q3 (≥5.42 to <19.04)	6.90	18,860	127	0.84 (0.69–1.03), *p* = 0.096	1.00 (0.81–1.22), *p* = 0.968
Q4 (≥19.04)	56.61	18,269	89	0.78 (0.62–0.99), *p* = 0.038	0.93 (0.73–1.19), *p* = 0.560
				*p* for trend = 0.095	*p* for trend = 0.640
Regular butter^#^					
Q1 (0)	0.00	35,545	277	Reference group	Reference group
Q2 (>0 to <0.82)	0.25	22,150	165	0.95 (0.79–1.16), *p* = 0.625	0.99 (0.81–1.20), *p* = 0.895
Q3 (≥0.82 to <3.76)	1.88	21,990	174	1.02 (0.84–1.23), *p* = 0.833	1.01 (0.84–1.23), *p* = 0.881
Q4 (≥3.76)	7.75	22,036	160	0.93 (0.77–1.14), *p* = 0.499	0.88 (0.72–1.08), *p* = 0.213
				*p* for trend = 0.570	*p* for trend = 0.191
Low-fat butter^#^					
Q1 (0)	0.00	85,311	663	Reference group	Reference group
Q2 (>0 to <0.37)	0.13	5577	34	0.76 (0.54–1.08), *p* = 0.126	0.83 (0.59–1.18), *p* = 0.303
Q3 (≥0.37 to <1.49)	0.76	5377	42	0.93 (0.68–1.26), *p* = 0.624	0.98 (0.72–1.34), *p* = 0.893
Q4 (≥1.49)	3.31	5456	37	0.78 (0.56–1.08), *p* = 0.135	0.80 (0.57–1.11), *p* = 0.180
				*p* for trend = 0.134	*p* for trend = 0.190
Regular cheese					
Q1 (<0.09)	0.00	25,808	179	Reference group	Reference group
Q2 (≥0.09 to <1.85)	0.74	26,914	181	1.11 (0.91–1.37), *p* = 0.303	1.08 (0.88–1.33), *p* = 0.454
Q3 (≥1.85 to <7.38)	3.85	24,892	194	1.09 (0.89–1.34), *p* = 0.409	1.02 (0.83–1.25), *p* = 0.874
Q4 (≥7.38)	13.78	24,107	222	1.19 (0.98–1.45), *p* = 0.087	1.11 (0.90–1.37), *p* = 0.336
				*p* for trend = 0.147	*p* for trend = 0.424
Low-fat cheese^#^					
Q1 (0)	0.00	55,534	461	Reference group	Reference group
Q2 (>0 to <0.89)	0.28	15,589	125	1.03 (0.84–1.25), *p* = 0.803	1.12 (0.91–1.37), *p* = 0.291
Q3 (≥0.89 to <3.07)	1.83	15,354	87	0.75 (0.59–0.94), *p* = 0.012	0.78 (0.62–0.98), *p* = 0.036
Q4 (≥3.07)	6.08	15,244	103	0.84 (0.68–1.04), *p* = 0.113	0.88 (0.71–1.09), *p* = 0.244
				*p* for trend = 0.051	*p* for trend = 0.109
No-fat cheese^#^					
Q1 (0)	0.00	76,813	603	Reference group	Reference group
Q2 (>0 to <0.47)	0.19	8688	58	0.90 (0.68–1.17), *p* = 0.430	0.96 (0.73–1.27), *p* = 0.790
Q3 (≥0.47 to <2.00)	1.01	8156	60	0.88 (0.68–1.15), *p* = 0.348	0.91 (0.70–1.19), *p* = 0.489
Q4 (≥2.00)	4.53	8064	55	0.84 (0.64–1.11), *p* = 0.216	0.85 (0.64–1.12), *p* = 0.252
				*p* for trend = 0.194	*p* for trend = 0.229

* *Adjusted for age (categorical), sex (male vs. female), race (White, Non-Hispanic vs. Other), body mass index at the time of enrollment (<25 kg/m^2^ vs. ≥25 kg/m^2^), education (≤ high school vs. ≥some college), smoking status (never vs. former ≤ 15 years since quit vs. former >15 years since quit vs. former year since quit unknown vs. current smoker ≤ 1 pack per day vs. current smoker >1 pack per day vs. current smoker intensity unknown), vegetable intake (continuous), fruit intake (continuous), tea intake (continuous), alcohol drinking status (never vs. former vs. current), total energy intake (continuous), randomization arm (intervention vs. control), family history of any cancer (yes vs. no), and marital status (married vs. not married)*.

# *Dairy products were categorized using non-consumers as the first category and tertiles of distribution for dairy products consumers*.

Among individual dairy foods, no statistically significant association was observed for a broad range of dairy products, including whole milk, 2% milk, 1% milk, skim milk, yogurt, regular butter, low-fat butter, regular cheese, low-fat cheese, and no-fat cheese (Table 2). These associations were not modified by smoking status (*p* for interaction >0.05).

## Discussion

In this large prospective study, various non-fermented and fermented dairy products were not associated with bladder cancer risk, in age- and sex-adjusted models or in multivariable-adjusted models. The null associations did not differ among strata defined by smoking status.

An updated WCRF-AICR Continuous Update Project in 2017 indicated that colorectal cancer risk decreased by 13% for each 400 g/day increment of dairy product consumption (95% CI: 10–17%) (17). Similarly, four recent meta-analyses (8, 11, 18, 19) reported an inverse association between intake of dairy products, especially fermented dairy foods and bladder cancer risk. These associations may differ by geographical region. For example, the meta-analysis by Mao et al. (19) reported a significant protective effect of milk consumption on bladder cancer in Asia but not in Europe. However, these meta-analysis included both case–control and cohort studies. Evidence from prospective studies was relatively limited and inconsistent (9, 10). Keszei et al. (9) reported that total dairy intake was not associated with bladder cancer risk in the Netherlands Cohort Study on Diet and Cancer, although there was some weak evidence that bladder cancer risk was inversely associated with fermented dairy products. Larsson et al. (10) also found no association between total dairy intake and bladder cancer risk in a prospective study of Swedish women and men. However, their study provided some evidence that a high intake of cultured milk may lower the risk of bladder cancer. Therefore, we undertook this analysis in PLCO study, and as a result, we found no evidence that intake of raw or fermented dairy was associated with the risk of bladder cancer.

Strengths of the PLCO study included the prospective design, large sample size, and high completeness of follow-up, which minimized the selection bias. Additionally, the information of main potential confounders for bladder cancer was available. Finally, we could analyze non-fermented and fermented milk separately and further perform analyses on dairy products with different fat contents. The limitations of PLCO included no repeated measurement of dietary product consumption during follow-up (the amount of dietary product consumption may change during follow-up), potential misclassification with self-reported questionnaire, and possible residual confounding (e.g., physical activity).

In summary, this study did not support the hypothesis that intake of dairy products was associated with the risk of bladder cancer. Further well-designed large prospective studies or collaborative studies are still warranted to verify our findings.

## Data Availability Statement

The datasets presented in this article are not readily available because the data that support the findings of this study are available from NIH PLCO study group. Restrictions apply to the availability of these data, which were used under license for this study. Requests to access the datasets should be directed to https://biometry.nci.nih.gov/cdas/datasets/plco/.

## Ethics Statement

The studies involving human participants were reviewed and approved by the Institutional Review Boards at the National Cancer Institute. The patients/participants provided their written informed consent to participate in this study.

## Author Contributions

XX designed the study, performed the data analyses, and drafted the manuscript. All authors contributed to the article and approved the submitted version.

## Conflict of Interest

The author declares that the research was conducted in the absence of any commercial or financial relationships that could be construed as a potential conflict of interest.
